# Patients With Pancreatitis‐Related Gene Variants Showed Higher Incidence of Hyperpancreatic Enzymemia After Endoscopic Retrograde Cholangiopancreatography

**DOI:** 10.1002/deo2.70320

**Published:** 2026-04-27

**Authors:** Daishi Kabemura, Toshio Fujisawa, Mitsuyoshi Suzuki, Ko Tomishima, Shigeto Ishii, Ippei Ikoma, Yasuhisa Jimbo, Muneo Ikemura, Hiroto Ota, Mako Ushio, Taito Fukuma, Sho Takahashi, Yusuke Takasaki, Daisuke Namima, Koichi Ito, Toshiaki Shimizu, Hiroyuki Isayama

**Affiliations:** ^1^ Department of Gastroenterology, Graduate School of Medicine Juntendo University Tokyo Japan; ^2^ Department of Pediatrics Juntendo University Faculty of Medicine Tokyo Japan; ^3^ Department of Medicine, Division of Gastroenterology, Faculty of Medicine Chulalongkorn University Bangkok Thailand

**Keywords:** endoscopic retrograde cholangiopancreatography, genetic susceptibility, pancreatitis‐related gene variants, post‐ERCP hyperpancreatic enzymemia, post‐ERCP pancreatitis

## Abstract

**Background:**

Post‐endoscopic retrograde cholangiopancreatography (post‐ERCP) pancreatitis (PEP) is the most severe adverse event associated with ERCP. Although numerous studies have identified risk factors for PEP, the role of genetic background in its development remains unexplored. The present study investigated the association between pancreatitis‐related gene variants (PRG‐variants) and PEP.

**Methods:**

This prospective, single‐center study included 94 patients with naïve papilla who underwent ERCP between October 2021 and August 2023. Targeted sequencing was performed to analyze variants in four PRGs: *PRSS1*, *SPINK1*, *CTRC*, and *CPA1*. Patients were classified into two groups based on the presence or absence of PRG‐variants, and the incidences of PEP and of post‐ERCP hyperpancreatic enzymemia (PEH) were compared.

**Results:**

PRG‐variants, regardless of their pathogenicity, were identified in 16 (17%) patients. Among all cases, PEP occurred in four (4%) patients, and PEH occurred in 27 (29%) patients. The incidence of PEP did not differ significantly with and without PRG‐variants (*p* = 0.532). However, the group with PRG‐variants had a significantly higher incidence of PEH, as demonstrated by both univariate (*p* = 0.013) and multivariate analyses (odds ratio, 6.291; 95% confidence interval, 1.133–34.934).

**Conclusions:**

The present pilot study suggests that patients with PRG‐variants, regardless of their pathogenicity, demonstrated a significantly higher incidence of PEH but not PEP. PEH may reflect pancreatic parenchyma injury and may progress to PEP when additional factors are present. Thus, PRG‐variants may contribute to biochemical pancreatic injury after ERCP. Further large‐scale studies and comprehensive genomic profiling of patients with PEH/PEP are warranted.

**Trial Registration:**

The central ethics committee approved the study protocol (M21‐0066).

## Graphical abstract

Pancreatitis‐related gene variants are associated with a higher incidence of post‐ERCP hyperpancreatic enzymemia, whereas no significant association is observed with post‐ERCP pancreatitis. These findings suggest that genetic background contributes to early biochemical pancreatic injury after ERCP and may help identify patients with increased susceptibility to procedure‐related pancreatic damage.

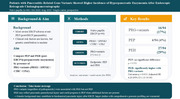

**Abbreviations**
CBD: common bile duct
CT: computed tomography
ERCP: endoscopic retrograde cholangiopancreatography
EUS‐BD: endoscopic ultrasound‐guided biliary drainage
NSAIDs: nonsteroidal anti‐inflammatory drugs
OR: odds ratio
PEH: post‐ERCP hyperpancreatic enzymemia
PEP: post‐ERCP pancreatitis
PRG: pancreatitis‐related gene
ULN: upper limit of normal.

## 1 Introduction

Endoscopic retrograde cholangiopancreatography (ERCP) is a widely performed diagnostic and therapeutic procedure. However, post‐ERCP pancreatitis (PEP) is a severe adverse event reported to occur in 2%–10% of cases [1]. Although most cases of PEP are mild, some can be fatal, with a reported mortality rate of approximately 0.7% [2]. Many studies have evaluated strategies for preventing PEP; however, its prevention remains challenging [3, 4]. Numerous studies have identified and categorized the risk factors for PEP into patient‐related and procedure‐related factors. Patient‐related factors include younger age, female, a history of PEP, suspected sphincter of Oddi dysfunction, a non‐dilated common bile duct (CBD), normal bilirubin levels, absence of chronic pancreatitis, and end‐stage renal disease. Procedure‐related factors include difficult cannulation, pancreatic guidewire passages, and pancreatic injections [5, 6, 7, 8, 9, 10]. Despite these findings, the exact cause of PEP remains unknown, and effective preventive measures have yet to be established. Among the identified risk factors, patient‐related factors play a significant role. Even when patients undergo the same endoscopic procedure, some develop neither PEP nor post‐ERCP hyperpancreatic enzymemia (PEH), while others develop severe PEP. The reason for this variability is not yet understood. By contrast, hereditary pancreatitis—characterized by recurrent pancreatitis episodes beginning in childhood—has been actively studied, leading to the identification of causative genes and genetic mutations [11, 12, 13, 14]. The frequency and severity of hereditary pancreatitis vary by gene and mutation type. However, the relationship between pancreatitis‐related genes (PRGs) and PEP remains unclear. PRG‐variants include both pathogenic and benign variants. In the present study, we investigated the influence of PRG‐variants regardless of their pathogenicity on PEH and PEP.

## 2 Methods

### 2.1 Study Design

This prospective observational study enrolled patients with a naïve papilla who underwent ERCP at Juntendo University Hospital between October 2021 and August 2023. The study was approved by the Juntendo University Clinical Research Review Board (approval number: M21‐0066), and written informed consent was obtained from all patients prior to the procedure. The present study adhered to the guidelines outlined in the revised Declaration of Helsinki [15].

Patients were excluded if they had a previous ERCP. Also excluded were patients scheduled to undergo endoscopic papillectomy, endoscopic ultrasound‐guided biliary drainage, endoscopic ultrasound‐guided pancreatic duct drainage, or percutaneous treatment, as well as those with acute pancreatitis at the time of ERCP or requiring emergent ERCP.

The sample size was determined for comparison of the incidence of PEP between patients with and without PRG‐variants. The expected prevalence of PRG‐variants was estimated based on previously published data in healthy controls, which reported variant frequencies of 4.3% for PRSS1, 2.76% for SPINK1, 1.2% for CTRC, and 0.1% for CPA1, with CFTR variants reported at 6.4% in a related study [16, 17, 18, 19]. Based on these data, the overall prevalence of variants was assumed to be 15%. Assuming a PEP incidence of 5% in the non‐PRG‐variants group (p0 = 0.05) and 30% in the PRG‐variants group (p1 = 0.30), with a one‐sided alpha of 0.05 and 80% power, the required total sample size was estimated to be 98 patients (15 patients with PRG‐variants and 83 patients without PRG‐variants).

### 2.2 Genetic Analysis

The targeted sequences of the four classical PRGs (*PRSS1*, *SPINK1*, *CTRC*, and *CPA1*) were analyzed using Sanger sequencing [20, 21]. DNA was extracted from peripheral blood leukocytes. Exon‐specific primers were designed based on published nucleotide sequences (*PRSS1*: NM_002769.3, *SPINK1*: NM_003122.3, *CTRC*: NM_007272.2, and *CPA1*: NM_001868.2) [11, 19, 22, 23]. Polymerase chain reaction amplification was performed using AmpliTaq Gold Polymerase (Applied Biosystems). PCR products were purified and subjected to cycle sequencing using the BigDye Terminator kit (Applied Biosystems). Sequencing was performed on a 3130 Genetic Analyzer (Applied Biosystems), and sequence data were analyzed using SeqScape version 2.5 software. Genetic analyses were performed after ERCP, and both endoscopists and outcome assessors were blinded to PRG status.

### 2.3 ERCP Procedures

Patients were sedated with intravenous pentazocine (15 mg) and midazolam (2–10 mg) before intubation, with continuous monitoring throughout ERCP. A TJF‐290 V duodenoscope (Olympus, Tokyo, Japan) or an ED‐580T duodenoscope (Fujifilm, Tokyo, Japan) was used, and biliary cannulation was attempted using an MTW catheter (MTW Endoskopie, Wesel, Germany). Guidewires, including the 0.025‐inch VisiGlide2 (Olympus), EndoSelector (Boston Scientific, Marlborough, MA, USA), and 0.035‐inch Seekmaster and Revowave Ultrahard (Piolax Medical Devices Inc., Kanagawa, Japan), were used for duct access and stent placement. For papillary manipulation, endoscopic sphincterotomy followed by papillary balloon dilation was performed using a CleverCut3 sphincterotome (Olympus) and either a 6‐ to 8‐mm Hurricane balloon (Boston Scientific) or REN balloon (Kaneka, Tokyo, Japan) [24]. Plastic stents or endoscopic nasobiliary drainage devices were placed for benign diseases or in preoperative settings, while fully covered self‐expandable metallic stents were used for malignant obstructions.

### 2.4 Clinical Data

Patient demographics and clinical characteristics (sex, age, body mass index, underlying disease, history of chronic pancreatitis, presence of periampullary diverticulum, Charlson comorbidity index, and CBD diameter) and laboratory data were collected. Serum amylase and lipase levels were measured 2 and 24 h after ERCP. Abdominal computed tomography (CT) was obtained only when abdominal pain or complications were suspected.

Procedural variables were documented, including prophylactic rectal nonsteroidal anti‐inflammatory drug (NSAIDs) use [25, 26, 27], cannulation success rate, procedure duration, difficult bile duct cannulation, cannulation time, number of contacts with the papilla, pancreatic duct cannulation, endoscopic papillary sphincterotomy, and pancreatic/biliary stent placement (including metallic stents).

### 2.5 Definitions and Outcomes

PEP was defined according to Cotton's criteria [28]. The primary outcome was PEP, and the secondary outcome was PEH. PEP was diagnosed when new or worsened pancreatic‐type abdominal pain occurred with serum amylase and lipase >3 × the upper limit of normal (ULN) at >24 h after ERCP.

PEH was defined as serum amylase and lipase levels >3 × ULN within 24 h after ERCP, including PEP cases.

Difficult bile duct cannulation was defined based on the European Society of Gastrointestinal Endoscopy Clinical Guideline using the “5‐5‐1” rule: cannulation time >5 min, >5 papillary contact, or ≥1 unintentional pancreatic duct cannulation [29, 30].

### 2.6 Statistical Analysis

Data were tabulated using Microsoft Excel (Microsoft, Redmond, WA, USA) and analyzed with SPSS Version 30 (IBM Corp., Armonk, NY, USA). Patient‐ and procedure‐related factors were compared using the χ2 test or the Mann–Whitney U test, as appropriate. Factors associated with the development of PEP and PEH were first evaluated by univariate analysis, and variables with a *p*‐value < 0.10 were entered into the multivariate model. To avoid multicollinearity and overfitting, variables judged to be clinically overlapping or strongly correlated were not entered simultaneously, and the final multivariate model was constructed using clinically relevant variables.

## 3 Results

### 3.1 Patient Characteristics and ERCP Procedure

**FIGURE 1:**
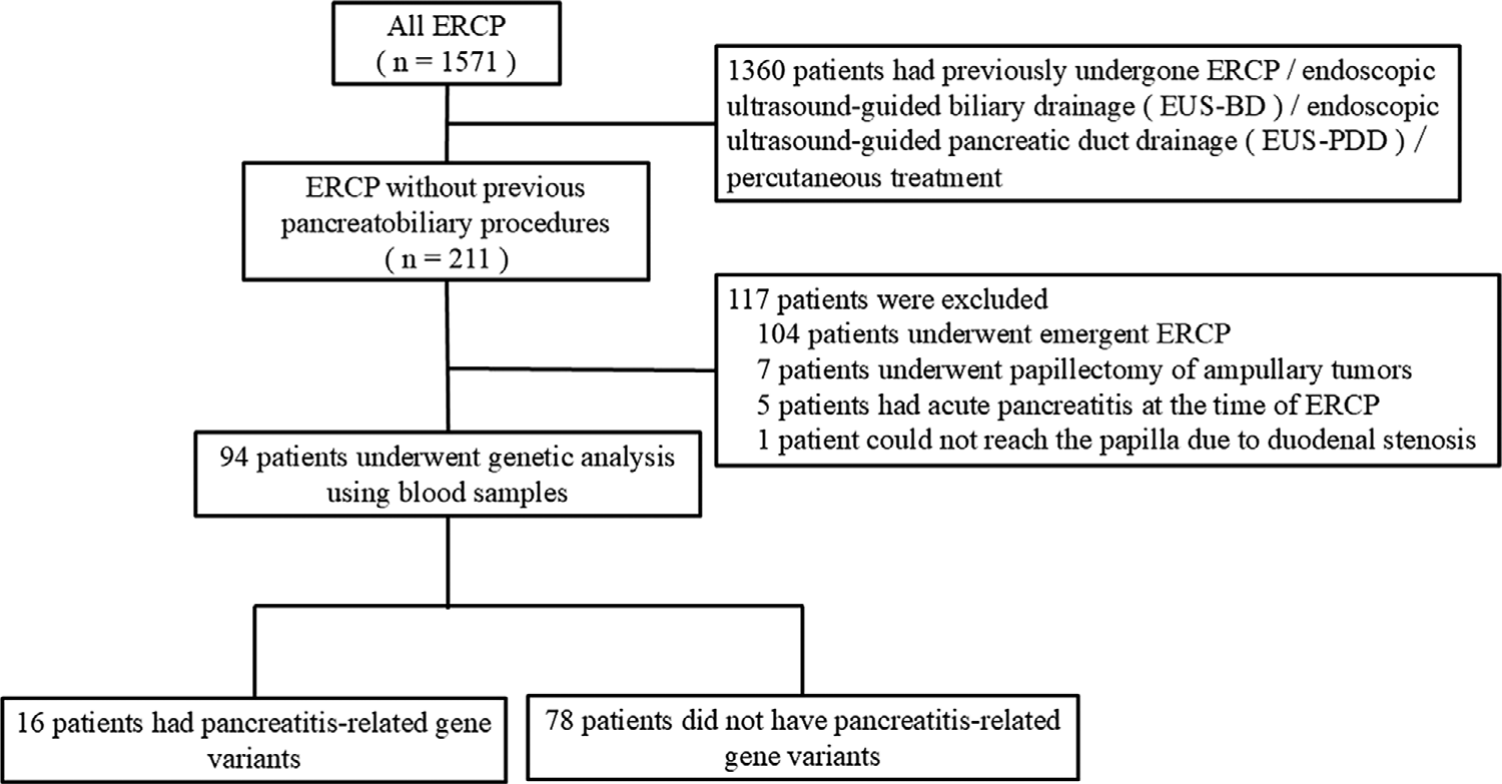
Flow chart of patient selection for the study.

A total of 1571 patients underwent ERCP between October 2021 and August 2023. After excluding 1360 with prior pancreatobiliary procedures and an additional 117 patients without prior procedure (lack of consent in 104 emergency cases, papillectomy in seven, acute pancreatitis in five, and duodenal stenosis in one), 94 patients were finally enrolled (Figure 1).

Patient characteristics and clinical outcomes are summarized in Table 1. The median age was 74 years; 21 (22%) patients were < 60 years, and 45 (48%) were women. Malignant obstructive jaundice was present in 29 (31%) patients (pancreatic cancer, 14; biliary tract cancer, 13; hepatocellular cancer, two), while 65 (69%) patients had benign diseases (bile duct stones, 55; benign bile duct strictures, four; pancreatic stones, three; benign pancreatic duct strictures, three).

**TABLE 1:** Patient characteristics and outcomes of endoscopic retrograde cholangiopancreatography (ERCP) procedure in all enrolled patients.

	Enrolled patients (*n* = 94)
**Patient characteristics**	
Age, y, median (IQR)	74 (62–79)
<60 years old, *n* (%)	21 (22)
Sex, *n* (%)	
Female	45 (48)
Male	49 (52)
BMI, kg/m^2^, median (IQR)	22.7 (19.5–25.5)
Patient disease, *n* (%)	
Malignancy	29 (31)
Pancreatic cancer	14
Biliary tract cancer	13
Hepatocellular cancer	2
Benign	65 (69)
Bile duct stone	55
Benign bile duct stricture	4
Pancreatic stone	3
Benign pancreatic duct stricture	3
History of chronic pancreatitis, *n* (%)	3 (3)
Periampullary diverticulum, *n* (%)	24 (26)
Charlson comorbidity index, median (IQR)	1 (0–3)
Rectal NSAIDs, *n* (%)	80 (85)
Baseline diameter of common bile duct, mm, median (IQR)	8.1 (5.9–13.7)
Baseline total bilirubin, mg/dL, median (IQR)	1.25 (0.74–4.39)
Baseline total bilirubin < 1.2 mg/dL, *n* (%)	46 (49)
Baseline creatinine, mg/dL, median (IQR)	0.72 (0.60–0.87)
Baseline amylase, U/L, median (IQR)	68 (51–95)
Baseline lipase, U/L, median (IQR)	42 (26–59)
Pancreatitis‐related gene variants, *n* (%)	16 (17)
**Outcomes of the ERCP procedure**	
Cannulation success, *n* (%)	92 (98)
Procedure time, min, median (IQR)	40 (26–57)
Difficult cannulation, *n* (%)	74 (80)
Cannulation time > 5 min, *n* (%)	58 (62)
Contacts with the papilla > 5 times, *n* (%)	32 (34)
Cannulation of pancreatic duct, *n* (%)	32 (39)
Endoscopic papillary sphincterotomy, *n* (%)	80 (85)
Pancreatic stent insertion, *n* (%)	12 (13)
Biliary stent insertion, *n* (%)	43 (46)
Metallic stent, *n* (%)	8 (9)
Post‐ERCP pancreatitis, *n* (%)	4 (4)
Post‐ERCP hyperpancreatic enzymemia, *n* (%)	27 (29)
Amylase, U/L, median (IQR)	
2 h	100 (59–161)
the next day	104 (59–205)
Lipase, U/L, median (IQR)	
2 h	59 (31–134)
the next day	47 (28–147)

Abbreviations: BMI, body mass index; ERCP, endoscopic retrograde cholangiopancreatography; IQR, interquartile range; NSAIDs, non‐steroidal anti‐inflammatory drugs.

NSAIDs were administered rectally before ERCP in 80 (85%) patients. The median bile duct diameter before ERCP was 8.1 mm. Blood test results before ERCP showed that the median serum bilirubin level was 1.25 mg/dL (below ULN in 46 (49%)). The median serum creatinine was 0.72 mg/dL, with one (1%) patient on hemodialysis.

Difficult cannulation occurred in 74 (80%) patients, including >5 min in 58 (62%) patients. Contact with the papilla occurred >5 times in 32 (34%) patients, and pancreatic duct cannulation was performed in 32 (39%) patients (all with pancreatography). Pancreatic stents were placed in 12 (13%) patients. PEP occurred in four (4%) patients (all mild), PEH in 27 (29%) patients.

### 3.2 Clinical Features of Patients With and Without PRG‐variants

**FIGURE 2:**
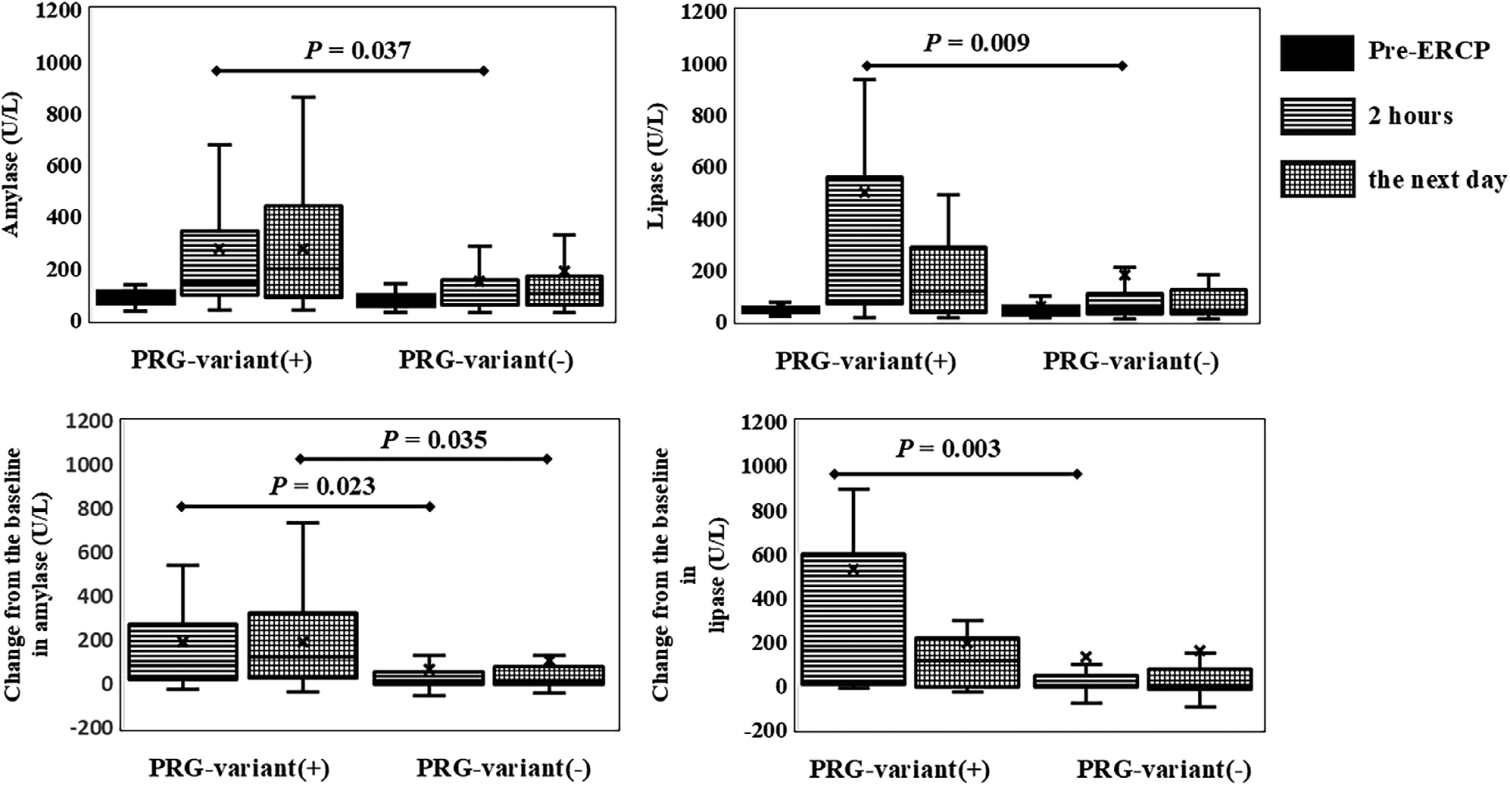
Serum pancreatic enzyme levels before and after endoscopic retrograde cholangiopancreatography (ERCP). (A) Serum amylase levels before ERCP, 2 h after ERCP, and the day after ERCP. (B) Serum lipase levels before ERCP, at 2 h after ERCP, and the day after ERCP. (C) Changes in serum amylase levels from baseline at 2 h after ERCP to the day after ERCP. (D) Changes in serum lipase levels from baseline at 2 h after ERCP to the day after ERCP. Values are presented as median with interquartile range.

PRG‐variants were identified in 16 (17%) patients. Details of the detected variants are shown in Table S1: *CPA1* in 9 patients, *PRSS1* in six, and *SPINK1* in one. No *CTRC* variants were detected, and no patients had multiple variants. In the PRG‐variants group, PEP occurred in one (6.3%) patient, and PEH was observed in nine (56%). The patient‐ and procedure‐related factors in patients with and without PRG‐variants are summarized in Table 2. Compared with patients without PRG‐variants, those with PRG‐variants were less often < 60 years (0% vs. 27%, *p* = 0.019) and more frequently underwent pancreatic duct cannulation (69% vs. 27%, *p* = 0.001), with no other significant differences. There was no significant difference in the incidence of PEP between the two groups (6.3% vs. 3.8%, *p* = 0.532) (Table 3), whereas PEH occurred more frequently in patients with PRG‐variants (56% vs. 23%, *p* = 0.013). Serum pancreatic enzyme levels were higher in patients with PRG‐variants, including 2‐h amylase (142 vs. 95 U/L, *p* = 0.037) and lipase (178 vs. 51 U/L, *p* = 0.009), as well as greater increases from baseline in amylase (2 h: 88 vs. 15 U/L, *p* = 0.023; next day: 127 vs. 14 U/L, *p* = 0.035) and lipase (2 h: 158 vs. 1 U/L, *p* = 0.003) (Figure 2).

**TABLE 2:** Patient characteristics and endoscopic retrograde cholangiopancreatography (ERCP) procedural results in patients with and without pancreatitis‐related gene (PRG) variants.

	Pancreatitis‐related gene variants group (*n* = 16)	Non‐pancreatitis‐related gene variants group (*n* = 78)	Univariate analysis *p‐*value
**Patient characteristics**			
Age, y, median (IQR)	77 (70–81)	73 (58–79)	0.087
<60 years old, *n* (%)	0 (0)	21 (27)	0.019
Sex, *n* (%)			0.852
Female	8 (50)	37 (47)	
Male	8 (50)	41 (53)	
BMI, kg/m^2^, median (IQR)	23.0 (21.0–23.5)	22.5 (19.3–25.8)	0.912
Patient disease, *n* (%)			0.375
Malignancy	3 (19)	26 (33)	
Benign	13 (81)	52 (67)	
History of chronic pancreatitis, *n* (%)	0 (0)	3 (4)	1.000
Charlson comorbidity index, median (IQR)	1 (0–2)	1 (0–3)	0.626
Periampullary diverticulum, *n* (%)	4 (25)	20 (26)	1.000
Rectal NSAIDs, *n* (%)	12 (75)	68 (87)	0.249
Baseline diameter of common bile duct, mm, median (IQR)	8.9 (6.8–14.9)	7.9 (5.9–12.8)	0.548
Baseline total bilirubin, mg/dL, median (IQR)	1.67 (0.83–2.81)	1.15 (0.74–4.61)	0.956
Baseline total bilirubin < 1.2 mg/dL, *n* (%)	6 (38)	40 (51)	0.315
Baseline creatinine, mg/dL, median (IQR)	0.74 (0.60–0.97)	0.72 (0.60–0.85)	0.528
Baseline amylase, U/L, median (IQR)	73 (62–111)	67 (51–93)	0.381
Baseline lipase, U/L, median (IQR)	42 (35–55)	43 (26–60)	0.665
**Results of the ERCP procedure**			
Cannulation success, *n* (%)	16 (100)	76 (97)	1.000
Procedure time, min, median (IQR)	43 (33–50)	40 (26–58)	0.651
Difficult cannulation, *n* (%)	13 (81)	61 (78)	0.623
Cannulation time > 5 min, *n* (%)	12 (75)	46 (59)	0.302
Contacts with the papilla > 5 times, *n* (%)	7 (44)	25 (32)	0.407
Cannulation of pancreatic duct, *n* (%)	11 (69)	21 (27)	0.001
Endoscopic papillary sphincterotomy, *n* (%)	14 (88)	66 (85)	1.000
Pancreatic stent insertion, *n* (%)	3 (19)	9 (12)	0.427
Biliary stent insertion, *n* (%)	9 (56)	34 (44)	0.377
Metallic stent, *n* (%)	1 (6)	7 (9)	1.000

Abbreviations: BMI, body mass index; ERCP, endoscopic retrograde cholangiopancreatography; IQR, interquartile range; NSAIDs, non‐steroidal anti‐inflammatory drugs.

**TABLE 3:** Post‐endoscopic retrograde cholangiopancreatography (post‐ERCP) pancreatitis and post‐ERCP hyperpancreatic enzymemia in patients with and without pancreatitis‐related gene (PRG) variants.

	Pancreatitis‐related gene variants group (*n* = 16)	Non‐pancreatitis‐related gene variants group (*n* = 78)	Univariate analysis *p*‐value
Post‐ERCP pancreatitis, *n* (%)	1 mild (6.3)	3 mild (3.8)	0.532
Post‐ERCP hyperpancreatic enzymemia, *n* (%)	9 (56)	18 (23)	0.013
Amylase, U/L, median (IQR)			
at 2 h	142 (97–343)	95 (57–154)	0.037
2 h—before ERCP	88 (20–244)	15 (‐2–57)	0.023
on the next day	199 (87–321)	100 (58–169)	0.087
the next day—before ERCP	127 (28–209)	14 (‐3–77)	0.035
Lipase, U/L, median (IQR)			
at 2 h	178 (69–323)	51 (31–105)	0.009
2 h—before ERCP	158 (6–473)	1 (‐8–43)	0.003
on the next day	115 (34‐269)	45 (28–119)	0.165
the next day—before ERCP	110 (‐5–214)	2 (‐16–73)	0.162

Abbreviations: ERCP, endoscopic retrograde cholangiopancreatography; IQR, interquartile range.

### 3.3 Relationship Between PEP and PRG‐Variants

Risk factors for PEP were compared between patients with and without PEP (Table 4 ). No significant difference was observed between the two groups regarding PRG‐variants (25% vs. 17%, *p* = 0.532). In univariate analyses, PEP was associated with age < 60 years (75% vs. 20%, *p* = 0.034) and lower serum bilirubin (0.63 vs. 1.37 mg/dL, *p* = 0.022); however, neither factor remained significant in the multivariate analysis (age <60: odds ratio [OR], 9.231; 95% confidence interval [CI] 0.836–101.940; *p* = 0.070; bilirubin: OR, 0.112; 95% CI 0.003–4.014; *p* = 0.230).

**TABLE 4:** Patient characteristics and results of endoscopic retrograde cholangiopancreatography (ERCP) procedure between the patients with and without pancreatitis (PEP).

	PEP (*n* = 4)	Non‐PEP (*n* = 90)	Univariate analysis *p*‐value	Multivariate analysis *p*‐value	Odds ratio (95% CI)
**Patient characteristics**					
Age, y, median (IQR)	44 (33–56)	75 (63–79)	0.002		
<60 years old, *n* (%)	3 (75)	18 (20)	0.034	0.070	9.231 (0.836–101.940)
Sex, *n* (%)			0.118		
Female	0 (0)	45 (50)			
Male	4 (100)	45 (50)			
BMI, kg/m^2^, median (IQR)	22.8 (21.9–24.5)	22.7 (19.5–25.5)	0.658		
Patient disease, *n* (%)			0.308		
Malignancy	0 (0)	29 (32)			
Benign	4 (100)	61 (68)			
History of chronic pancreatitis, *n* (%)	0 (0)	3 (3)	1.000		
Charlson comorbidity index, median (IQR)	1 (0–2)	1 (0–3)	0.698		
Periampullary diverticulum, *n* (%)	0 (0)	24 (27)	0.569		
Rectal NSAIDs, *n* (%)	4 (100)	76 (84)	1.000		
Baseline diameter of common bile duct, mm, median (IQR)	7.5 (6.3–8.8)	8.1 (5.9–13.9)	0.552		
Baseline total bilirubin, mg/dL, median (IQR)	0.63 (0.50–0.79)	1.37 (0.81–4.53)	0.022	0.230	0.112 (0.003–4.014)
Baseline total bilirubin < 1.2 mg/dL, *n* (%)	4 (100)	42 (47)	0.054		
Baseline creatinine, mg/dL, median (IQR)	0.75 (0.72–0.79)	0.72 (0.59–0.87)	0.602		
Baseline amylase, U/L, median (IQR)	66 (57–76)	68 (47–97)	0.805		
Baseline lipase, U/L, median (IQR)	32 (27–34)	44 (26–62)	0.125		
Pancreatitis‐related gene variants, *n* (%)	1 (25)	15 (17)	0.532		
**Results of the ERCP procedure**					
Cannulation success, *n* (%)	4 (100)	88 (98)	1.000		
Procedure time, min, median (IQR)	47 (39–62)	40 (25–57)	0.358		
Difficult cannulation, *n* (%)	2 (50)	72 (80)	0.240		
Cannulation time > 5 min, *n* (%)	3 (75)	55 (61)	1.000		
Contacts with the papilla > 5 times, *n* (%)	1 (25)	31 (34)	1.000		
Cannulation of pancreatic duct, *n* (%)	3 (75)	29 (32)	0.116		
Endoscopic papillary sphincterotomy, *n* (%)	3 (75)	77 (86)	0.458		
Pancreatic stent insertion, *n* (%)	1 (25)	11 (12)	0.430		
Biliary stent insertion, *n* (%)	1 (25)	42 (47)	0.621		
Metallic stent, *n* (%)	0 (0)	8 (9)	1.000		

Abbreviations: BMI, body mass index; CI, confidence interval; ERCP, endoscopic retrograde cholangiopancreatography; IQR, interquartile range; NSAIDs, non‐steroidal anti‐inflammatory drugs; OR, odds ratio; PEP, post‐ERCP pancreatitis.

### 3.4 Relationship Between PEH and PRG‐Variants

Risk factors for PEH were compared between patients with and without PEH, as summarized in Table 5. PEH was associated with a higher prevalence of PRG‐variants (33% vs. 10%, *p* = 0.013), lower baseline total bilirubin (0.87 vs. 1.66 mg/dL, *p* = 0.001) with more patients having normal bilirubin (<1.20 mg/dL; 74% vs. 39%, *p* = 0.002), higher baseline amylase (82 vs. 64 U/L, *p* = 0.011), more frequent pancreatic duct cannulation (70% vs. 19%, *p* < 0.001), and less frequent biliary stent insertion (30% vs. 52%, *p* = 0.040). In multivariate analysis, PRG‐variants (OR 6.291, 95% CI 1.133–34.934), normal baseline bilirubin (OR 6.679, 95% CI 1.590–28.056), and pancreatic duct cannulation (OR 7.292, 95% CI 1.924–27.634) remained significant, whereas baseline amylase and biliary stent insertion did not. In addition, to evaluate potential confounding by procedure‐related factors, stratified analyses according to pancreatic duct cannulation status were performed. Within each stratum, PEH was compared between patients with and without PRG‐variants. The incidence of PEH was numerically higher in patients with PRG‐variants than in those without PRG‐variants both among patients with pancreatic duct cannulation (72.7% [8/11] vs. 52.4% [11/21]; *p* = 0.450) and among those without pancreatic duct cannulation (20.0% [1/5] vs. 12.3% [7/57]; *p* = 0.511), but neither difference reached statistical significance (Figure S1).

**TABLE 5:** Patient characteristics and results of endoscopic retrograde cholangiopancreatography (ERCP) procedure between the patients with and without post‐ERCP hyperpancreatic enzymemia (PEH).

	PEH (*n* = 27)	Non‐PEH (*n* = 67)	Univariate analysis *p*‐value	Multivariate analysis *p*‐value	Odds ratio (95% CI)
**Patient characteristics**					
Age, y, median (IQR)	71 (60–80)	75 (63–79)	0.525		
<60 years old, *n* (%)	7 (26)	14 (21)	0.596		
Sex, *n* (%)			0.624		
Female	14 (52)	31 (46)			
Male	13 (48)	36 (54)			
BMI, kg/m^2^, median (IQR)	22.6 (19.7–24.4)	22.8 (19.5–25.8)	0.871		
Patient disease, *n* (%)			0.077	0.324	3.138 (0.324–30.444)
Malignancy	4 (15)	25 (37)			
Benign	23 (85)	42 (63)			
History of chronic pancreatitis, *n* (%)	1 (2)	2 (3)	1.000		
Charlson comorbidity index, median (IQR)	1 (0–3)	1 (0–3)	0.943		
Periampullary diverticulum, *n* (%)	7 (26)	17 (25)	0.956		
Rectal NSAIDs, *n* (%)	21 (78)	59 (88)	0.216		
Baseline diameter of common bile duct, mm, median (IQR)	8.1 (6.5–11.1)	8.0 (5.8–14.7)	0.742		
Baseline total bilirubin, mg/dL, median (IQR)	0.87 (0.55–1.40)	1.66 (0.88–5.87)	0.001		
Baseline total bilirubin < 1.2 mg/dL, n (%)	20 (74)	26 (39)	0.002	0.01	6.679 (1.590–28.056)
Baseline creatinine, mg/dL, median (IQR)	0.72 (0.61–1.05)	0.73 (0.60–0.84)	0.358		
Baseline amylase, U/L, median (IQR)	82 (67–107)	64 (46–89)	0.011	0.599	1.003 (0.992–1.015)
Baseline lipase, U/L, median (IQR)	44 (34–70)	41 (26–58)	0.233		
Pancreatitis‐related gene variants, n (%)	9 (33)	7 (10)	0.013	0.035	6.291 (1.133–34.934)
**Results of the ERCP procedure**					
Cannulation success, *n* (%)	27 (100)	65 (97)	1.000		
Procedure time, min, median (IQR)	45 (33–68)	40 (22–52)	0.086	0.481	1.009 (0.984–1.034)
Difficult cannulation, *n* (%)	21 (78)	53 (84)	0.55		
Cannulation time > 5 min, *n* (%)	17 (63)	41 (61)	0.921		
Contacts with the papilla > 5 times, *n* (%)	13 (48)	19 (28)	0.083		
Cannulation of pancreatic duct, *n* (%)	19 (70)	13 (19)	<0.001	0.003	7.292 (1.924–27.634)
Endoscopic papillary sphincterotomy, *n* (%)	22 (81)	58 (87)	0.512		
Pancreatic stent insertion, *n* (%)	10 (37)	2 (3)	<0.001		
Biliary stent insertion, *n* (%)	8 (30)	35 (52)	0.040	0.107	0.234 (0.040–1.367)
Metallic stent, *n* (%)	2 (7)	6 (9)	1.000		

Abbreviations: BMI, body mass index; CI, confidence interval; ERCP, endoscopic retrograde cholangiopancreatography; IQR, interquartile range; NSAIDs, non‐steroidal anti‐inflammatory drugs; OR, odds ratio; PEH, post‐ERCP hyperpancreatic enzymemia.

## 4 Discussion

The present study investigated the influence of PRG‐variants on the development of PEP and PEH, to help elucidate its causes. Among the 94 patients analyzed, PRG‐variants were identified in 16 (17%) (Table S1). PEP was observed in a patient with *PRSS1* p.Thr137Met/Thr, while PEH was associated with variants in the *PRSS1*, *CPA1*, and *SPINK1* genes. The pathogenic significance of PRG‐variants differs by mutation, and some variants remain of uncertain significance [31]. Previous studies have reported an association between PEP and mutations in the interferon regulatory factor 2 binding protein 1 gene (*IRF2BP1*) and gamma‐glutamyl transferase 1 gene (*GGT1*) [32, 33], but their pathological significance remains unclear. In the present study, three mutations were classified as pathogenic based on ClinVar (https://www.ncbi.nlm.nih.gov/clinvar/): *PRSS1* p.Gly208Ala/Gly (Patients 7 and 11, Table S1), *CPA1* p.Pro140Leu/Pro (Patient 14), but none of them developed PEP. In addition, patient‐related factors, procedure‐related factors, and clinical outcomes were compared between the pathogenic group (*n* = 3) and the non‐pathogenic group (*n* = 91), which included patients with non‐pathogenic PRG‐variants and those without PRG‐variants (Table S2). No significant differences were observed between the two groups. Specifically, PEP occurred in 0% of the pathogenic group and 4.4% of the non‐pathogenic group (p = 1.000), while PEH occurred in 33.3% and 28.6%, respectively (*p* = 1.000). In comparing patient characteristics between cases with and without PEP, no significant differences were observed in the prevalence of PRG‐variants. However, when patients were divided into those with and without PEH, the number of patients with PRG‐variants was significantly higher in the PEH group. Logistic regression analysis further supported the association between PRG‐variants and the development of PEH.

PEH is defined as a transient elevation of serum pancreatic enzymes following ERCP, differing from PEP in the absence of abdominal pain. However, abdominal pain is subjective and varies among individuals. Uchino et al. reported that “hidden PEP” (painless pancreatitis) is present in 37% of patients with PEH when computed tomography is used to screen for adverse events [34].

In the present study, CT was obtained only when abdominal pain or complications were suspected; therefore, hidden PEP could not be systematically assessed. Nevertheless, multiple studies have suggested that elevations of amylase and/or lipase within a few hours after ERCP are associated with subsequent PEP and can be used for early prediction [35, 36]. Accordingly, we measured pancreatic enzymes at 2 and 24 h after ERCP and regarded PEH as an early biochemical phenotype reflecting pancreatic stress or injury, which may have pathophysiological continuity with clinically overt PEP in a subset of patients, although PEH is not synonymous with PEP.

Regarding biological plausibility, PRG‐variants may increase vulnerability of the pancreatic parenchyma to minor mechanical stimulation or hydrostatic pressure load during ERCP through dysregulation of intrapancreatic trypsin activity and impaired protective responses against acinar injury. *PRSS1* variants are known to promote excessive activation of trypsinogen and/or suppress trypsin degradation, whereas *SPINK1* variants reduce the inhibitory capacity against trypsin; in addition, *CPA1* variants have been reported to be associated with acinar cell injury mediated by endoplasmic reticulum stress. These mechanisms may predispose genetically susceptible patients to enzyme elevation (PEH), and with additional procedure‐related factors or inflammatory triggers, PEH may progress to clinically overt PEP. In the present study, pancreatic duct cannulation was more frequent among patients who developed PEH; however, stratified analyses according to pancreatic duct cannulation status showed a similar direction of association between PRG‐variants and PEH within each stratum, although the differences did not reach statistical significance due to the limited sample size.

From a clinical perspective, the immediate significance of predicting PEH alone may be limited. However, pre‐procedural identification of patients carrying genetic variants potentially associated with PEP may allow more vigilant post‐procedural monitoring and consideration of intensified preventive strategies, such as rectal NSAID administration, prophylactic pancreatic duct stenting, selection of EUS‐guided biliary drainage (EUS‐BD) instead of ERCP in selected cases, or early termination of ERCP, which may contribute to reducing the risk of PEP.

The present study had several limitations. First, the incidence of PEP among patients with PRG‐variants was 6.3%, which was lower than the 30% anticipated at the study design stage; as a result, the sample size may have been relatively insufficient, limiting the ability to examine thoroughly the relationship between PEP and PRG‐variants. Second, as an observational study, potential biases between groups could not be fully excluded. Third, the present study was limited to examining only four classical PRG‐variants because the clinically available panel at our institution was restricted at the time of study planning. Finally, all detected PRG‐variants were analyzed collectively regardless of their pathogenicity; given the small number of pathogenic or likely pathogenic variants, stratified analysis according to variant classification was not statistically feasible, and variant classification may change over time with re‐annotation. Variant reclassification over time has been reported in clinical genetic testing [37]. In conclusion, the relationship between PRG‐variants and PEP remains inconclusive. However, patients with PRG‐variants were more likely to develop PEH following ERCP, suggesting that PRG‐variants may represent a patient‐related factor associated with early biochemical pancreatic injury. Larger studies with broader genetic analyses are needed to validate these findings and clarify clinical utility.

## Author Contributions

**Daishi Kabemura**, **Toshio Fujisawa**, and **Mitsuyoshi Suzuki**: conception of the work, literature review, interpretation of the data, and drafting of the article. **Ippei Ikoma**, **Yasuhisa Jimbo**, **Muneo Ikemura**, and **Hiroto Ota**: obtaining informed consent and collecting the data. **Mako Ushio**, **Taito Fukuma**, **Sho Takahashi**, **Yusuke Takasaki**, **Daisuke Namima**, **Koichi Ito**, **Ko Tomishima**, and **Shigeto Ishii**: collection of the data and interpretation of the results. **Toshiaki Shimizu** and **Hiroyuki Isayama**: critical revision of the article for important intellectual content and supervision of the work.

## Funding

This work was supported by JSPS KAKENHI Grant Number 24K10340.

## Conflicts of Interest

Hiroyuki Isayama received research grants from Boston Scientific Japan and FUJIFILM Corporation.

## Ethics Statement

Approval of the research protocol by an Institutional Review Board.

## Consent

Written informed consent was obtained from all patients prior to the procedure.
